# Increased frequency and nocturia in a middle aged male may not always be due to Benign Prostatic Hypertrophy (BPH): a case report

**DOI:** 10.1186/1757-1626-2-168

**Published:** 2009-10-27

**Authors:** Kumar Gaurav, Jamie Fitch, Mukta Panda

**Affiliations:** 1 Department of Medicine, University of Tennessee, College of Medicine, Chattanooga, TN, USA

## Abstract

Primary signet ring cell carcinoma of urinary bladder is a rare type of bladder tumor and carries a very high mortality rate. It may have a clinical presentation similar to common diseases like Benign Prostatic Hypertrophy (BPH) and the management options are extremely limited. We report a case of 58 year old Caucasian male who presented with a 5 month history of increased frequency of urination, nocturia and weight loss without any fever or hematuria. He was found to have an increased creatinine of 2.8 mg/dl and a prostate specific antigen level of 0.18 ng/ml. His azotemia was thought to be secondary to BPH. A foley catheter was initially placed with a plan for outpatient follow up. On removal of the catheter his problems persisted and he returned to the hospital. Diagnostic work up including abdominal ultrasonography, computed tomography (CT) scan, retrograde pyelogram, cystography and cystoscopic biopsies revealed the diagnosis of primary signet ring cell carcinoma of urinary bladder. Although cystectomy was planned, our patient passed away before this could be done.

## Introduction

Primary signet ring cell carcinoma of urinary bladder is a rare type of bladder tumor and carries a very high mortality rate. It may have a clinical presentation and symptomatology similar to common diseases like Benign Prostatic Hypertrophy (BPH). It is diagnosed with bladder biopsy. Unfortunately, due to the rarity of this disease entity and due to the aggressive nature of tumor, the treatment options are extremely limited.

## Case Presentation

**Figure 1:**
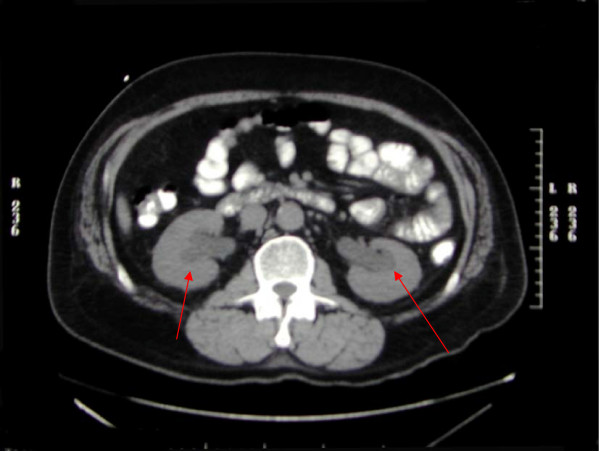
**Computerized tomography scan showing bilateral hydronephrosis and hydroureter**.

**Figure 2:**
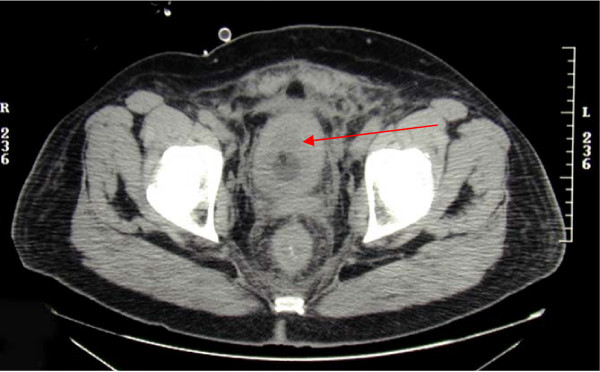
**Computerized tomography scan showing urinary bladder wall thickening**.

**Figure 3:**
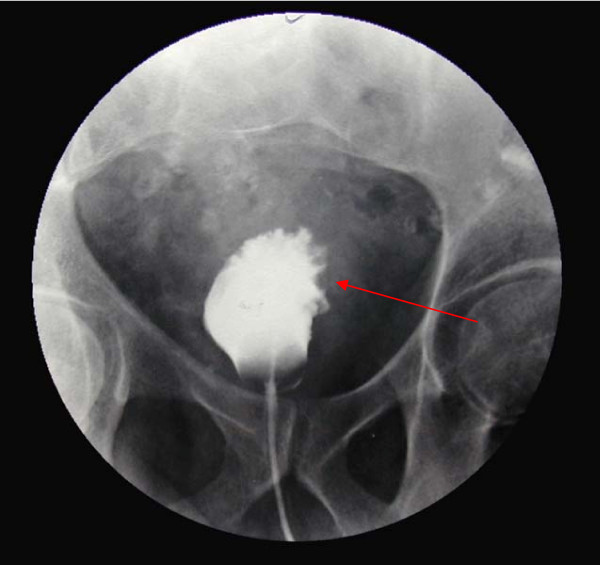
**Cystogram showing marked trabecular thickening**.

**Figure 4:**
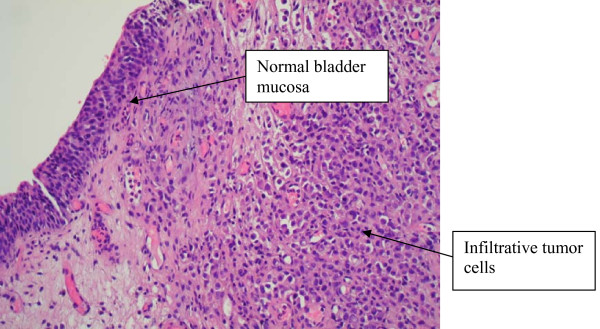
**Bladder biopsy slide showing normal transitional epithelium on the left and infiltrative tumor cells on the right**.

**Figure 5:**
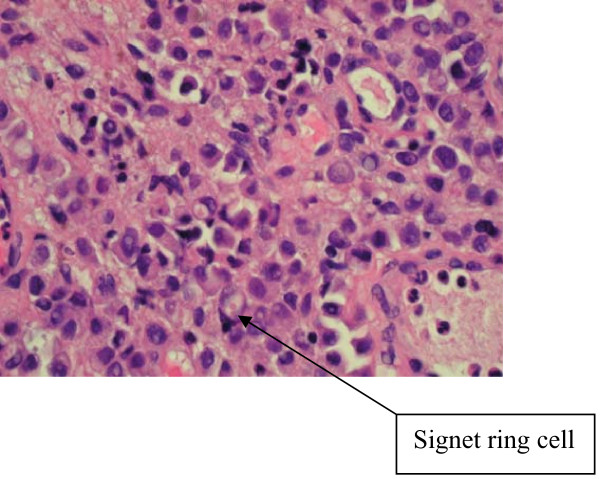
**High power field of bladder biopsy showing tumor cells, many with signet ring appearance**.

**Figure 6:**
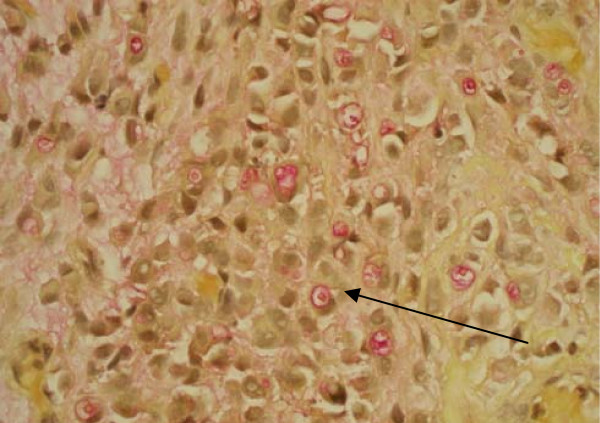
**Mucicarmine stain showing mucin vacuoles in signet ring cells**.

A 58 year old previously healthy Caucasian male presented with a five month history of increased frequency of urination, feeling of incomplete emptying and nocturia. He denied any history of fever, hematuria, nausea, vomiting or diarrhea. He complained of approximate 10 pound weight loss over a period of 1 week. He reported a previous diagnosis of enlarged prostate with a normal Prostate Specific Antigen (PSA). He also reported a recent history of "small heart attack" for which he was medically treated at an outlying facility where he was also informed about poor kidney function. He also gave history of recently diagnosed hypertension and a 80 pack year of ongoing smoking. His medications included amlodipine, finasteride, doxazosin, metoprolol, clopidrogel, aspirin and lovastatin. All of these were started 5 days prior to presentation. Physical examination revealed a healthy appearing male with normal vital signs. His rectal exam revealed a slightly enlarged, non-tender prostate. Rest of the physical exam was unremarkable. Laboratory data revealed hemoglobin 10.3 g/dl, blood urea nitrogen 24 mg/dl, creatinine 2.7 mg/dl, and PSA level 0.18 ng/ml. Urinalysis was negative for RBC's, WBC's, bacteria, or nitrates. Retroperitoneal ultrasound showed normal sized kidneys with mild pyelocaliectasis bilaterally, prostate measured 4 × 2.7 × 2.6 cm with homogenous echotexture, otherwise unremarkable. Immediately after placement of foley catheter he had 650 ml of urine output. Blood urea nitrogen decreased to 18 mg/dl and serum creatinine decreased to 1.7 mg/dl. His azotemia was thought to be secondary to BPH and he was discharged with indwelling foley catheter to be followed up as an outpatient. At outpatient clinic, on removal of his foley catheter, his post void residual was found to be 150 ml. Various management options including surgical interventions were discussed with the patient in detail. After discussion patient was discharged home to be followed up as an outpatient but was started on tamsulosin. He returned to the hospital with complaints of urgency and frequency. His creatinine had increased to 5.0 mg/dl. Abdominal CT at this time revealed bilateral hydronephrosis and hydroureter (Figure 1 and 2). This finding was confirmed on retrograde pyelogram. Cystography showed marked thickening of the urinary bladder trabeculae (Figure 3). Cystoscopy revealed the entire bladder mucosa to be thickened and edematous with an exaggerated granular type appearance and bilateral uretero-vesical junction stenosis prompting placement of bilateral ureteral stents. He subsequently had a diuresis producing 6 liters of urine. This was followed by a reduction in the serum creatinine from 5.2 to 3.1 mg/dl over two days. Pathological analysis of tissue from random bladder wall biopsies at the time of cystoscopy revealed an infiltrate of cells beneath the surface epithelium (Figure 4). These cells were described as small, with a high nuclear-cytoplasmic ratio. Many cells showed a cytoplasmic vacuolization with displacement of crescentic, hyperchromatic nuclei (Figure 5). Special stain results were positive for mucin (Figure 6), pan-cytokeratin, CK7 and CK20 but negative for PSA and PAP (prostatic acid phosphatase). Based on these results, a diagnosis of Primary Signet Ring Cell Carcinoma (PSRCC) of the bladder was established. He underwent esophagogastroduodenoscopy and colonoscopy to evaluate possible primary site of his malignancy but were found to be negative. Management plans were then made for radical cystectomy. But the subsequent course was complicated by colitis secondary to Clostridium difficile requiring total colectomy with diverting ileostomy. Pathologic evaluation of the removed colon showed no evidence of malignant involvement. Recovery was further complicated by myocardial infarction requiring coronary artery bypass grafting. The patient failed to recover and continued to deteriorate. After discussion with family his care was transferred to hospice and he passed away. Cystectomy was therefore not performed.

## Discussion

Primary signet-ring cell carcinoma (PSRCC) is a rare variation of adenocarcinoma of the urinary bladder. English literature review on pubmed reveals less than 100 cases reported. Adenocarcinoma constitutes 2% of all bladder cancers with the majority being metastatic rather than primary[1]. A study of 713 cases of primary bladder tumor in Sweden revealed 4 signet ring cell carcinomas, only 0.6% [2]. Another series of 715 bladder cancers in Germany revealed 18 adenocarcinomas but only one with the classic histologic pattern of signet-ring cell carcinoma [3]. Signet-ring cells are much more commonly described in primary adenocarcinoma of the stomach, colon, breast or gallbladder and malignancies of these organs should be excluded before diagnosing PSRCC of urinary bladder [3]. A 3:1 male to female predominance has been shown [4], but in male patients, it is especially necessary to rule out prostatic adenocarcinoma as a possible site of origin [5].

Most patients present in middle-age with symptoms indistinguishable from the much more common transitional cell carcinoma of the bladder. The most common presenting symptoms are hematuria and difficulty in urination which can be mistaken for a urinary tract infection. In cases of rapid growth in the trigone area, oliguria, bladder irritation, and renal failure can be the initial presenting signs [6]. Yamamoto and associates described one patient, similar to our own, who presented with renal failure and oliguria without gross hematuria [7]. Cases have also been reported presenting with a palpable suprapubic mass [8] and one with a palpable supraclavicular lymph node [4]. The average time from initial symptoms to the first physician visit has been reported to be five months, [8] but no relationship between the duration of symptoms before presentation and the stage of disease has been found[5].

Grignon et al compared the gross features of 34 cases of primary signet-ring cell carcinoma. They found that 47.1% have no mucosal or mass lesion present on cystoscopy. The most common description is of "edematous mucosa" but descriptions of erythematous or finely granular mucosa are also found. As in our case, the bladder wall is often described as thickened or fibrotic [4]. Signet-ring cells first invade the mucosa and submucosa of a hollow organ with eventual full-thickness involvement. This pattern of invasion can produce extensive lateral spread without the development of a protruding neoplasm, [9] but in more than half of cases, a definite mass lesion is found with morphology ranging from polypoid or pedunculated to sessile to ulcero-infiltrative [4]. Often a pyelogram will show a filling defect or the CT scan may show diffuse bladder wall thickening [1]. Because this tumor mainly has an infiltrative rather than exophytic growth pattern, it is not readily identifiable on cystoscopy. A full-thickness biopsy of the bladder may be necessary to make the diagnosis [8]. As in our patient, this diffusely infiltrating lesion can occlude the ureters early in its course causing obstruction and hydronephrosis [9].

Signet ring cells are described as crescent shaped cells containing nuclei compressed to one edge of the cell by large amounts of cytoplasmic mucin appearing as a single clear vacuole in some tumors and as a foamy, multivesicular cytoplasmic material in others [4,8]. Mucin accumulations form in the cytoplasm and nuclei are unevenly distributed [7]. Routine mucin staining of otherwise normal transitional cell carcinoma will reveal signet ring cells in many cases and the exact percentage of signet ring cells that must be present in order to make the diagnosis of PSRCC of the bladder has not been established. Holmang and associates suggested that 50-60% of the tumor should be made up of signet-ring cells to make this classification [2]. However, because the linitis plastica-like pattern of diffuse signet-ring cell infiltration is associated with a poorer prognosis, it has been suggested that only this pattern should be considered a pure signet-ring cell carcinoma [4]. Bladder tumors found to consist solely of signet-ring cells should prompt a thorough search for a distant primary site. Those consisting of a mix of signet-ring cells and transitional cells are more likely to be of primary bladder origin [1]. Also, PSRCC is usually a solitary lesion (63%) in contrast to transitional cell carcinoma which is most often multifocal (66%) [8].

Three theories for the histogenesis of this type of carcinoma have been suggested in literature. First is the metaplastic potential of urothelium which may occur along the surface of the bladder or in areas of cystitis cystica within the bladder. The second is that diffuse signet ring cell adenocarcinoma derives from isolated signet ring cells that exist scattered in normal transitional epithelium. The third possibility is that of signet ring cell carcinoma arising from metaplastic transitional cell carcinoma [4,10-14].

Treatment options for PSRCC are limited. This is a rare disease entity; no specific chemotherapy has been recommended in the literature. Radiotherapy has also not been shown to be successful [2]. Total cystectomy with excision of adjacent tissues may offer some hope in this aggressive malignancy [8]. There is no specific serum tumor marker for PSRCC of the bladder, but elevated carcinoembryonic antigen (CEA) levels have been reported. In cases where CEA is elevated at diagnosis, levels may be used for post-operative monitoring [7]. Death is most often the result of distant metastases. Therefore, the most successfully reported treatment strategy is resection of the primary tumor before metastasis occurs [1]. As extensive intramural growth is so often found at diagnosis, total cystectomy may be the only hope for cure [15]. Kondo and associates reported no difference in mean remission rates between patients who received radiotherapy either pre- or post-operatively and those who did not [8]. In 2001, Yasuhiro and associates described successful treatment of a PSRCC of the bladder with intra-arterial administration of carboplatin through the left vesicular artery. Complete remission had been maintained for 44 months at the time of their publication [16]. Unfortunately, this is the only such case reported. This disease typically carries a poor prognosis, often due to extensive spread at the time of diagnosis.

## Conclusion

PSRCC of urinary bladder is a rare entity with a very poor prognosis. Since patients may present with clinical picture similar to common diseases like BPH it may be difficult to diagnose early in the course of the disease. Treatment options are extremely limited and not well studied. Therefore it is important for the clinicians to be aware of this disease entity.

## Consent

The patient is now deceased. Written informed consent for publication of this manuscript and any accompanying images was obtained from the patient's next of kin. A copy of the written approval is available for review by the Editor-in-Chief of this Journal.

## Competing interests

The authors declare that they have no competing interests.

## Authors' contributions

KG and JF participated in patient management, literature review and helped in conceptualization and drafting of manuscript. MP helped in conceptualization, drafting and reviewing of the manuscript. All authors read and approved the final manuscript
